# Optimal surgeon and hospital volume thresholds to reduce mortality and length of stay for CABG

**DOI:** 10.1371/journal.pone.0249750

**Published:** 2021-04-14

**Authors:** Ying-Yi Chou, Juey-Jen Hwang, Yu-Chi Tung

**Affiliations:** 1 Institute of Health Policy and Management, National Taiwan University, Taipei, Taiwan; 2 Division of Cardiology, Department of Internal Medicine, National Taiwan University Hospital Yun-Lin Branch, Dou‑Liu City, Taiwan; 3 Division of Cardiology, Department of Internal Medicine, National Taiwan University Hospital, Taipei, Taiwan; Providence VA Medical Center, UNITED STATES

## Abstract

**Objective:**

We used nationwide population-based data to identify optimal hospital and surgeon volume thresholds and to discover the effects of these volume thresholds on operative mortality and length of stay (LOS) for coronary artery bypass surgery (CABG).

**Design:**

Retrospective cohort study.

**Setting:**

General acute care hospitals throughout Taiwan.

**Participants:**

A total of 12,892 CABG patients admitted between 2011 and 2015 were extracted from Taiwan National Health Insurance claims data.

**Main Outcome Measures:**

Operative mortality and LOS. Restricted cubic splines were applied to discover the optimal hospital and surgeon volume thresholds needed to reduce operative mortality. Generalized estimating equation regression modeling, Cox proportional-hazards modeling and instrumental variables analysis were employed to examine the effects of hospital and surgeon volume thresholds on the operative mortality and LOS.

**Results:**

The volume thresholds for hospitals and surgeons were 55 cases and 5 cases per year, respectively. Patients who underwent CABG from hospitals that did not reach the volume threshold had higher operative mortality than those who received CABG from hospitals that did reach the volume threshold. Patients who underwent CABG with surgeons who did not reach the volume threshold had higher operative mortality and LOS than those who underwent CABG with surgeons who did reach the volume threshold.

**Conclusions:**

This is the first study to identify the optimal hospital and surgeon volume thresholds for reducing operative mortality and LOS. This supports policies regionalizing CABG at high-volume hospitals. Identifying volume thresholds could help patients, providers, and policymakers provide optimal care.

## Introduction

The volume-outcome relationship has been established for various surgeries, including cardiac and noncardiac specialty surgeries. For coronary artery bypass surgery (CABG), previous studies have found that patients who received CABG from high-volume hospitals or surgeons had lower operative mortality [1,2] and length of stay (LOS) [3,4]. Recently, many studies have explored volume thresholds for surgeries to improve quality of care [5,6]. For CABG, Gutacker et al. suggested an annual hospital volume threshold of 415 cases for decreasing the risk of in-hospital 30-day death [7]. However, the study did not identify the optimal hospital and surgeon volume for decreasing operative mortality or explore the impact of hospital and surgeon volume on operative mortality and LOS. To date, there has been no empirical research identifying optimal hospital and surgeon volume thresholds to decrease operative mortality and LOS.

Most of the volume-outcome studies rely on adjusting for observable covariates, but potential bias in volume-effect estimates may emerge because we do not consider self-selection into hospitals based on volume and unobserved covariates, such as a patient’s health risk [8,9]. If sicker patients prefer to receive surgery at high-volume hospitals, the volume benefits may be underestimated. In contrast, the volume benefits may be overestimated if healthier patients self-select into high-volume hospitals. The previous studies, which identified the volume thresholds and explored the volume-outcome relationship, only adjusted for observable covariates and did not account for self-selection bias related to unobservable covariates [7,10].

In Taiwan, the National Health Insurance Administration (NHIA) introduced the national health insurance (NHI) scheme in March 1995. The NHI scheme is a single-payer system that provides universal health coverage to Taiwan’s 23.5 million residents, and almost all of the hospitals and clinics have contracts with the NHIA. Patients can freely select a hospital where they receive CABG. A single, prospectively determined fixed bundled payment for CABG was implemented in Taiwan, and it includes all services provided by the hospital, surgeon, and other practitioners during the entire inpatient stay. A closed-staff system is carried out by hospitals in Taiwan, which allows surgeons employed by hospitals to treat patients admitted to their hospitals and treat their outpatients. Public reporting and selective referral are not available for patients in Taiwan, so they cannot use public quality data and cannot obtain recommendations from a referral physician to select better-performing providers. Moreover, a regionalization or centralization policy for CABG care has not been developed, so Taiwan’s healthcare delivery system may not influence the volume-outcome relationship for CABG.

This study, using nationwide, population-based data from Taiwan, discovered optimal hospital and surgeon volume thresholds for reducing operative mortality and LOS for patients with CABG. We also examined the effects of hospital and surgeon volume thresholds on the operative mortality and LOS of CABG.

## Methods

### Data source

We used the national research database, which is provided by the Health and Welfare Data Science Center in Taiwan. The deidentified secondary database contained the following files: NHI inpatient medical claims, outpatient medical claims, NHI beneficiaries, medical facilities, and death certificate. The protocol for this study was approved by the Institutional Review Board of the National Taiwan University Hospital (protocol # 201804039RINA). The requirement of informed consent was waived because the dataset we used in this study was deidentified secondary data.

### Study population

Patients who received CABG (International Classification of Diseases, 9th Revision, Clinical Modification [ICD-9-CM] procedure code 36.1–36.2) (N = 16,398) [11] from acute care hospitals between 2011 and 2015 were considered the study population. We excluded patients with the following criteria: patients who were transferred out (N = 172), patients younger than 18 years (N = 10), patients who received subsequent CABG between 2011 and 2015 (N = 41), patients without information on sex (N = 39), patients without information on low-income status (N = 106) and residential location (N = 66), and patients without information on the hospital accreditation level (N = 61). Moreover, we also excluded patients with valve surgery (N = 3,011) during the index admission. There were 12,892 patients who received CABG from 507 surgeons in 73 hospitals.

### Dependent variables

The primary outcome was all-cause operative mortality, which was defined as death from any cause before hospital discharge or within 30 days of hospitalization [1,2,12]. The operative mortality is considered to be a comprehensive mortality measure by the Society of Thoracic Surgeons and the National Quality Forum [13].

The secondary outcomes was LOS, which is an indicator of resource usage and is a proxy of efficiency [14]. Length of stay was computed by subtracting the day of the index admission from the day of hospital discharge.

### Independent variables

For each CABG, hospital volume was defined as the total number of CABG procedures carried out by that hospital in the calendar year before the year of the patient’s admission [15], using unique hospital identification numbers. Surgeon volume was defined as the total number of CABG performed by that surgeon in the calendar year before the year of the patient’s admission using unique surgeon identification numbers. Based on the hospital and surgeon volume thresholds, we divided hospital and surgeon volumes into high-volume and low-volume groups.

### Covariates

After reviewing the related literature, we included several patient, surgeon, and hospital covariates that might have influenced the risk of adverse outcomes after CABG admission. The patient characteristics included sex, age, low-income status, in-hospital treatments, history of percutaneous coronary intervention (PCI), comorbid conditions, medical history, and traveling distance [11,16–18].

The Charlson-Deyo index was applied to calculate each patient’s comorbidities [19]. The index is defined as the sum of the weighted scores, covering 17 medical conditions during the previous year, and the index admission. The comorbidity burden corresponds to the increases of the scores. Moreover, we adjusted the use of internal mammary artery (IMA) revascularization and the use of cardiopulmonary bypass during the index CABG procedure. Regarding PCI history, we considered whether a patient received PCI for 365 days before the index CABG procedure. Regarding medical history, we adjusted the following medical conditions during the previous year based on the diagnosis codes: acute myocardial infarction (ICD-9-CM codes: 410, 412), congestive heart failure (ICD-9-CM codes: 398.91, 402.01, 402.11, 402.91, 404.01, 404.03, 404.11, 404.13, 404.91, 404.93, 425.4–425.9, 428), peripheral vascular disease (ICD-9-CM codes: 093.0, 437.3, 440, 441, 443.1–443.9, 47.1, 557.1, 557.9, V43.4), hypertension (ICD-9-CM codes: 401–405), diabetes mellitus (ICD-9-CM codes: 250), renal dysfunction (ICD-9-CM codes: 580–586), and chronic obstructive pulmonary disease(ICD-9-CM codes: 490–496) [11,16,17,19]. The traveling distance was calculated using zip codes from the patient residence and the hospital where patients received CABG [18].

The surgeon characteristics included age. The hospital characteristics included size (small/large), geographic location (Taipei, northern, central, southern, Kao-Ping, eastern), and urban/rural area. We divided hospital size into two equal groups based on the number of beds. The hospitals located in a rural area were classified as rural hospitals according to the definition of urbanization established by Taiwan’s National Health Research Institutes [20,21]. We did not include hospital accreditation and teaching status as covariates because almost all patients receiving CABG in high-volume hospitals were treated in higher accreditation hospitals and teaching hospitals.

### Statistical analysis

Restricted cubic splines (RCSs) were applied to explore the nonlinear relationship between provider volume and risk-standardized operative mortality for CABG [10,15,22–31]. The risk adjustment methods recommended by the Agency for Healthcare Research and Quality were used to calculate the risk-standardized operative mortality. We used a generalized estimating equation logistic regression model to compute the expected operative mortality for each hospital and surgeon after adjusting the patient-level, surgeon-level, and hospital-level covariates. The risk-standardized operative mortality was defined as the ratio of observed mortality to expected mortality multiplied by the national unadjusted operative mortality [32]. Because the distribution of risk-standardized operative mortality was skewed, we performed a log transformation to make the distribution less skewed.

In the RCS analysis, 7 knots were selected for the hospital volume, and 4 knots were applied to the surgeon volume, according to the minimum Akaike information criterion [15,23,25,31,33]. Because we identified a range of inflection points from the nonlinear relationship between hospital or surgeon volume and the log of risk-standardized operative mortality rates, the receiver operating characteristic (ROC) curve for the multivariable logistic regression was applied to identify the optimal volume [15,23,24]. The ROC curve was used to evaluate the ability of provider volume to discriminate between patients with death and those without death. The area under the ROC curve summarizes the location of the ROC curve and describes the validity of hospital and surgeon volumes [34]. The range of area under the curve is from 0.5 (no discrimination) to 1.0 (outstanding discrimination).

Generalized estimating equation regression and Cox proportional-hazards modeling as well as instrumental variable (IV) estimation were used to explore the effects of hospital and surgeon volume thresholds on operative mortality and LOS after adjusting for observed covariates and unobserved covariates. We applied an IV approach to address the endogeneity of independent variables due to unobserved variables, such as health risks [9]. The models that were not adjusted for unobserved variables were labeled naïve models.

For the IV approach, we applied two-stage residual inclusion (2SRI) estimation, which would not be biased because of the nonlinearity of the regression model [35]. The IV is used to estimate the treatment effects (provider volume) accounting for self-selection into a treatment according to unobserved covariates. The IVs had to meet the following assumptions: (1) the IV was strongly related to treatment selection; (2) the IV did not influence outcomes directly or correlate with unobserved covariates. Differential-distance was used as an IV [9,36,37]. We defined the IV as the distance to the nearest hospital and the distance to the nearest high-volume hospital [38].

The 2SRI estimations were inferred from the generalized estimating equation models. The first stage regressed the hospital volume threshold, including the IV, surgeon volume threshold, and all covariates, and obtained the residual terms. The second stage added these residual terms into generalized estimating equation logistic regression models for operative mortality and into Cox proportional-hazards model with a robust sandwich variance estimate for LOS [39]. The Cox proportional-hazards regression model is recommended for modeling LOS because the logarithmic (or other) transformations of LOS and other right skewed data is not useful for policy making [40], and the presence of competing events (such as death) may affect LOS [39]. Because the event in this survival analysis was discharge from hospital, the effect size of each factor on discharge was evaluated by regression coefficient. The hazard ratio (HR) was calculated by taking the exponential of regression coefficient. A HR less than 1 indicated that the probability of discharge is lower, and a HR greater than 1 implied that the probability of discharge is higher. In other words, patients with longer LOS had a lower probability of discharge. SAS software, version 9.4 (SAS Institute) was used for the analysis. All statistical tests were 2-tailed and used a type I error rate of 0.05.

## Results

Table 1 presents the descriptive statistics. Of all patients, 78.2% were male, and 47.8% were above 65 years of age. Most patients received cardiopulmonary bypass (64.6%) and IMA revascularization (70.1%) during the index CABG procedure, and had hypertension (25.9%) and diabetes mellitus (59.5%) during the previous year and index admission. The mean Charlson score was 3.3 (standard deviation [SD] 2.4). The mean traveling distance was 22.6 kilometers (km) (SD 45.6). In total, 65.5% were admitted to academic medical centers, 99.7% were admitted to teaching hospitals, and 94.6% were admitted in an urban area. The mean annual hospital volume was 149 cases (SD 113), and the mean annual surgeon volume was 44 cases (SD 37). The all-cause operative mortality was 8.2%. The mean LOS was 21.6 days (SD 19.9).

**Table 1:** Study population characteristics and unadjusted patient outcome.

Variable	N	%	Mean	SD	Median (IQR)
No. patients	12,892	100.0	-	-	-
Patient Characteristics					
Male	10,076	78.2	-	-	-
Age, y					
18–49	1,188	9.2	-	-	-
50–64	5,534	42.9	-	-	-
65–79	5,211	40.4	-	-	-
80+	959	7.4	-	-	-
Low income	249	1.9	-	-	-
In-hospital treatment					
Cardiopulmonary bypass	8,330	64.6	-	-	-
IMA	9,038	70.1	-	-	-
Surgical history					
PCI	1,576	12.2	-	-	-
Charlson score	-	-	3.3	2.4	3 (1–5)
Medical history					
Acute myocardial infarction	5,219	40.5	-	-	-
Congestive heart failure	4,820	37.4	-	-	-
Peripheral vascular disease	1,152	8.9	-	-	-
Hypertension	3,345	25.9	-	-	-
Diabetes	7,669	59.5	-	-	-
Renal dysfunction	3,565	27.7	-	-	-
COPD	2,381	18.5	-	-	-
Traveling distance, km	-	-	22.6	45.6	17 (12–24)
Surgeon Characteristics					
Surgeon volume	-	-	43.8	36.7	33 (16–60)
2011	-	-	49.9	40.5	36 (20–71)
2012	-	-	44.0	37.5	35 (16–60)
2013	-	-	42.0	35.1	33 (14–58)
2014	-	-	43.5	35.5	34 (15–65)
2015	-	-	39.1	33.4	28 (16–52)
Age, y					
≦40	3,356	26.0	-	-	-
41–50	5,754	44.6	-	-	-
51+	3,782	29.3	-	-	-
Hospital Characteristics					
Hospital volume	-	-	148.6	113.4	129 (56–213)
2011	-	-	157.8	114.2	160 (61–212)
2012	-	-	143.3	108.7	133 (56–213)
2013	-	-	148.9	107.9	129 (53–221)
2014	-	-	155.7	125.7	101 (57–255)
2015	-	-	137.0	108.5	99 (54–206)
Accreditation level					
Academic medical center	8,448	65.5	-	-	-
Regional	4,383	34.0	-	-	-
District	61	0.5	-	-	-
Teaching	12,857	99.7	-	-	-
Location					
Taipei	6,444	50.0	-	-	-
Northern	1,398	10.8	-	-	-
Central	1,982	15.4	-	-	-
Southern	1,476	11.4	-	-	-
Kao-Ping	1,298	10.1	-	-	-
Eastern	294	2.3	-	-	-
Size					
Non-large	6,455	50.1	-	-	-
Large	6,437	49.9	-	-	-
Urban	12,194	94.6	-	-	-
Patient outcome					
Operative mortality	1,060	8.2	-	-	-
Length of stay, d	-	-	21.6	19.9	8 (12–24)

COPD indicates chronic obstructive pulmonary disease; IMA, internal mammary artery; IQR, interquartile range; PCI, percutaneous coronary intervention; SD, standard deviation.

**Fig 1:**
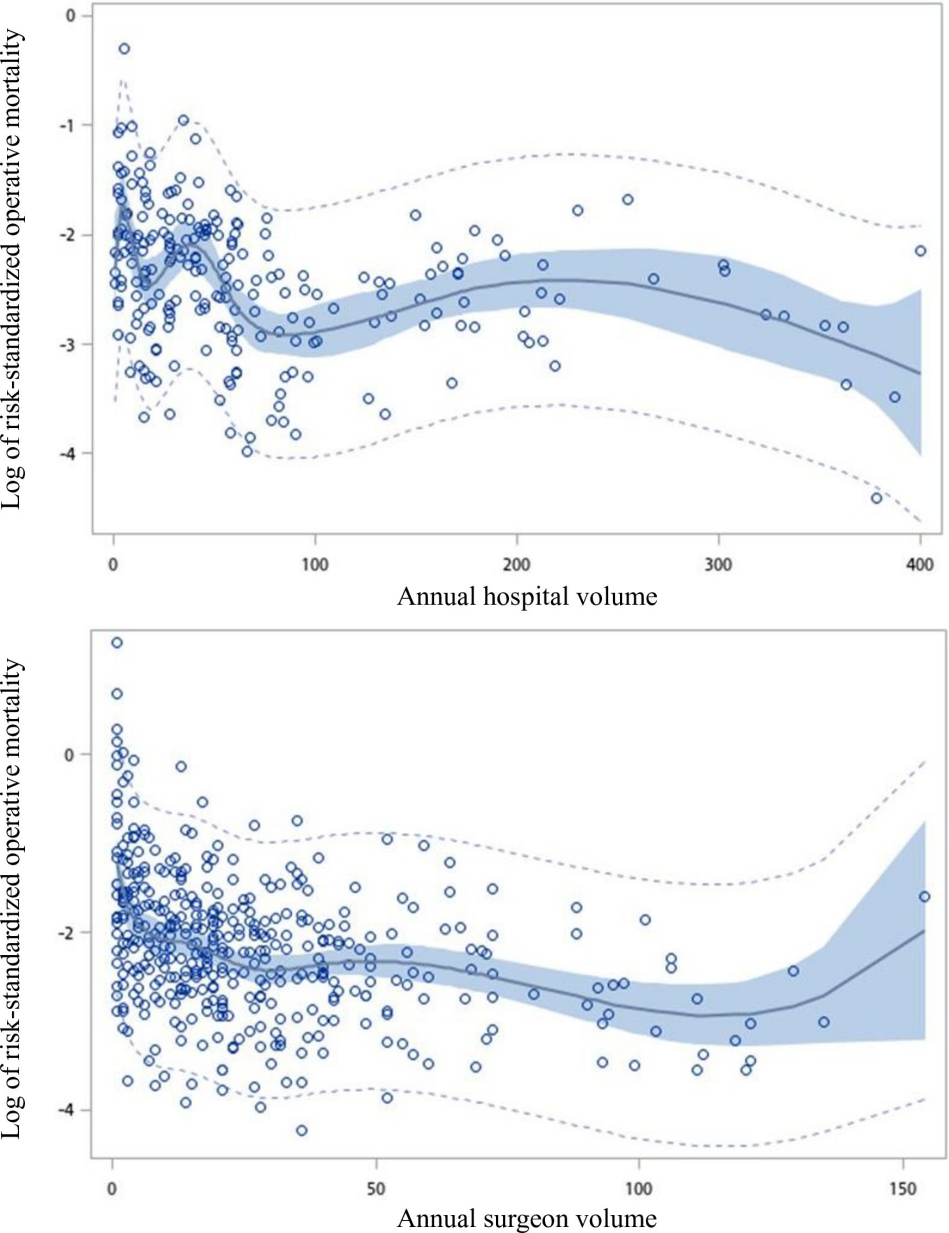
Restricted cubic spline plot of log of risk-standardized operative mortality rate versus the annual number of coronary artery bypass graft per hospital and per surgeon. The light dotted curves represent the 95% confidence intervals about the predicted operative mortality rate. The dark curve represents the regression line. The shaded areas represent the 95% confidence intervals about the regression line.

There was a negative nonlinear relationship, which was demonstrated by the restricted cubic splines of hospital and surgeon volumes vs the log of the operative mortality (Fig 1). Increasing hospital and surgeon volumes were associated with decreasing of the log of the operative mortality up to 55 and 5 cases per year, respectively, after which operative mortality continued to decline at a lower rate with increasing hospital and surgeon volume. ROC curves were calculated relating the annual hospital volume (with cutoff points ranging from 40 to 155 cases a year) and annual surgeon volume (with cutoff points ranging from 5 to 120 cases a year) to the operative mortality (S1 and S2 Tables). According to the maximum area under the curves, the optimal cutoff points for hospital and surgeon volumes were 55 and 5 cases per year, respectively.

Pearson chi-squared or t-tests revealed relationship of lower hospital and surgeon volumes to higher operative mortality and longer LOS (Table 2). Patients receiving CABG from low-volume hospitals were more likely to have operative mortality compared with patients receiving CABG from high-volume hospitals (13.5% vs 6.6%). Patients receiving CABG from low-volume surgeons were more likely to have operative mortality (21.2% vs 6.9%) and longer LOS (31.5 days vs 20.6 days) compared with patients receiving CABG from high-volume surgeons.

**Table 2:** Comparison of patient, surgeon, and hospital characteristics by hospital and surgeon volume.

Variable	Hospital volume			Surgeon volume		
	Low (<55 cases/y)	High (≧55 cases/y)	P	Low (<5 cases/y)	High (≧5 cases/y)	P
No. patients	3,105	9,787		1,198	11,694	
Patient Characteristics						
Male (%)	76.3	78.7	0.004	75.4	78.4	0.014
Age, y (%)						
18–49	8.2	9.5	0.031	8.5	9.3	<0.001
50–64	42.5	43.1		38.6	43.4	
65–79	41.1	40.2		43.0	40.2	
80+	8.2	7.2		9.9	7.1	
Low income (%)	2.7	1.7	<0.001	2.8	1.8	0.017
In-hospital treatment						
Cardiopulmonary bypass (%)	68.1	63.5	<0.001	66.9	64.4	0.076
IMA (%)	57.1	74.2	<0.001	53.6	71.8	<0.001
Surgical history						
PCI (%)	11.7	12.4	0.297	12.0	12.2	0.820
Charlson score, mean (SD)	3.4 (2.4)	3.3 (2.4)	0.053	3.6 (2.5)	3.3 (2.3)	<0.001
Median (IQR)	3 (2–5)	3 (1–5)		3 (2–5)	3 (1–5)	
Medical history						
Acute myocardial infarction (%)	38.1	41.2	0.002	45.6	40.0	<0.001
Congestive heart failure (%)	39.9	36.6	<0.001	43.2	36.8	<0.001
Peripheral vascular disease (%)	8.0	9.2	0.028	10.4	8.8	0.056
Hypertension (%)	28.6	25.1	<0.001	33.1	25.2	<0.001
Diabetes (%)	61.0	59.0	0.049	61.1	59.3	0.232
Renal dysfunction (%)	29.7	27.0	0.004	33.9	27.0	<0.001
COPD (%)	19.6	18.1	0.067	18.5	18.5	0.954
Traveling distance, km, mean (SD)	14.6 (31.8)	25.2 (48.9)	<0.001	17.2 (35.0)	23.2 (46.6)	<0.001
Median (IQR)	5 (0–15)	9 (0–24)		6 (0–18)	8 (0–22)	
Surgeon Characteristics						
Volume (%)						
High	79.9	94.1	<0.001	-	-	-
Low	20.1	5.9		-	-	
Age, y (%)						
≦40	40.6	21.4	<0.001	67.4	21.8	<0.001
41–50	42.1	45.4		22.4	46.9	
51+	17.3	33.2		10.2	31.3	
Hospital Characteristics						
Accreditation level (%)						
Academic medical center	14.4	81.8	<0.001	43.5	67.8	<0.001
Regional	83.6	18.2		55.5	31.8	
District	2.0	0.0		1.0	0.4	
Teaching (%)	98.9	100.0	<0.001	99.7	99.7	0.663
Location (%)						
Taipei	29.5	56.5	<0.001	31.1	51.9	<0.001
Northern	9.1	11.4		21.5	9.7	
Central	22.2	13.2		17.4	15.2	
Southern	22.7	7.9		9.3	11.7	
Kao-Ping	12.0	9.5		14.6	9.6	
Eastern	4.5	1.5		6.1	1.9	
Size (%)						
Non-large	83.1	39.6	<0.001	61.9	48.9	<0.001
Large	16.9	60.4		38.1	51.1	
Urban (%)	81.2	98.8	<0.001	87.9	95.3	<0.001
Patient outcome						
Operative mortality (%)	13.5	6.6	<0.001	21.2	6.9	<0.001
Length of stay, d, mean (SD)	21.2 (21.4)	21.7 (19.4)	0.188	31.5 (40.8)	20.6 (16.0)	<0.001
Median (IQR)	17 (12–24)	17 (13–24)		21 (14–36)	17 (12–23)	

COPD indicates chronic obstructive pulmonary disease; IMA, internal mammary artery; IQR, interquartile range; PCI, percutaneous coronary intervention; SD, standard deviation.

Table 3 presents descriptive statistics for hospitals and surgeons. During 2011 and 2015, the number of low-volume hospitals ranged from 37 (63.8%) to 44 (69.8%), and the number of low-volume surgeons ranged from 92 (44.0%) to 121 (51.5%). The percentage of high-volume hospitals located in the urban area ranged from 90.5% to 100.0%, and the percentage of low-volume hospitals located in the urban area ranged from 73.8% to 83.8%. The mean annual hospital volume declined from 63 cases per year to 53 cases per year, and the mean annual surgeon volume declined from 17 cases per year to 15 cases per year. The risk-standardized operative mortality for hospitals ranged from 6.3% to 9.7%, and the risk-standardized operative mortality for surgeons ranged from 4.4% to 7.7%.

**Table 3:** Operative mortality and characteristics of hospitals and surgeons.

Variable	2011	2012	2013	2014	2015
No. hospitals	58	61	63	64	65
Volume					
Mean (SD)	62.6 (82.1)	57.1 (78.0)	55.2 (74.3)	56.8 (81.0)	53.3 (73.1)
Median (IQR)	30 (8–71)	21 (9–70)	28 (4–59)	26 (8–67)	28 (8–62)
No. high-volume hospitals	21	21	19	22	22
%	36.2	34.4	30.2	34.4	33.8
Urban	19	21	18	22	22
%	90.5	100.0	94.7	100.0	100.0
No. low-volume hospitals	37	40	44	42	43
%	63.8	65.6	69.8	65.6	66.2
Urban	31	32	36	31	34
%	83.8	80.0	81.8	73.8	79.1
Crude operative mortality					
Mean (SD)	13.5 (40.1)	8.4 (10.6)	20.0 (37.3)	8.7 (14.3)	11.5 (19.0)
Median (IQR)	4.9 (0–10.3)	5.3 (0–11.1)	6.7 (0–17.6)	4.9 (0–11.8)	4.9 (0–13.0)
Risk-standardized operative mortality					
Mean (SD)	6.3 (5.4)	7.6 (8.0)	9.7 (11.4)	8.9 (7.9)	7.4 (7.3)
Median (IQR)	6.2 (0–9.6)	5.8 (0–10.2)	7.3 (0–10.5)	9.0 (0–13.6)	6.5 (0–11.2)
No. surgeons	209	227	235	229	225
Volume					
Mean (SD)	17.1 (25.1)	14.9 (23.8)	14.0 (22.1)	15.0 (22.9)	14.7 (21.6)
Median (IQR)	9 (0–23)	4 (0–21)	4 (0–20)	5 (0–20)	6 (0–22)
No. high-volume surgeons	117	109	114	118	123
%	56.0	48.0	48.5	51.5	54.7
No. low-volume surgeons	92	118	121	111	102
%	44.0	52.0	51.5	48.5	45.3
Crude operative mortality					
Mean (SD)	7.1 (19.6)	11.1 (32.2)	7.6 (20.6)	7.4 (20.7)	9.5 (24.8)
IQR	0.0–5.7	0.0–7.1	0.0–4.8	0.0–5.3	0.0–6.1
Risk-standardized operative mortality					
Mean (SD)	4.4 (9.3)	5.8 (11.0)	6.4 (24.9)	7.7 (20.0)	6.2 (14.3)
IQR	0.0–7.1	0.0–8.2	0.0–6.7	0.0–8.4	0.0–8.2

IQR indicates interquartile range; SD, standard deviation.

Table 4 demonstrates the results of the naïve model and the instrumental variable analyses. For the 2SRI model, we used the distance to the nearest hospital and the distance to the nearest high-volume hospital as the instrumental variables. In the first stage of 2SRI estimates, the instrumental variables were related to the hospital volume, which was identical to the assumption of the IV. The second stage of the 2SRI model showed a negative association of hospital volume and surgeon volume with operative mortality. Surgeon volume was associated with LOS. Patients admitted to low-volume hospitals had 309% higher odds of operative mortality (odds ratio 4.09, 95% confidence interval [CI] 1.33–12.55) than those admitted to high-volume hospitals. Patients treated by low-volume surgeons had 69% higher odds of operative mortality (odds ratio 1.69, 95% CI 1.21–2.38) than those treated by high-volume surgeons. Moreover, patients treated by low-volume surgeons had 36% lower risk of discharge (hazard ratio 0.64, 95% CI 0.56–0.72) than those treated by high-volume surgeons, which meant that patients treated by low-volume surgeons had a higher risk of prolonged hospitalization. In addition, patient age, IMA use during the same hospitalization, medical history, surgeon age, hospital location and size were related with operative mortality and discharge.

**Table 4:** The first stage regression of instrumental variable model and the effects of hospital volume and surgeon volume on operative mortality and discharge.

Variable	High-volume hospital			Operative mortality			Discharge		
	OR	(95% CI)	P	OR*	(95% CI)	P	HR*	(95% CI)	P
Naïve model									
Hospital volume with < 55 cases a year (ref: high volume)	-	-	-	1.90	(1.42–2.55)	<0.001	0.90	(0.81–1.00)	0.057
Surgeon volume with < 5 cases a year (ref: high volume)	-	-	-	1.90	(1.38–2.62)	<0.001	0.63	(0.56–0.70)	<0.001
2-stage residual inclusion model									
Stage 1									
IV: Distance to nearest hospital, km	1.01	(1.00–1.02)	0.038	1.00	(0.99–1.00)	0.564	-	-	-
IV: Distance to nearest high-volume hospital, km	0.99	(0.98–1.00)	0.035	-	-	-	1.00	(1.00–1.00)	0.833
Stage 2^a^									
Hospital volume with < 55 cases a year (ref: high volume)	-	-	-	4.09	(1.33–12.55)	0.014	0.80	(0.50–1.28)	0.350
Surgeon volume with < 5 cases a year (ref: high volume)	-	-	-	1.69	(1.21–2.38)	0.002	0.64	(0.56–0.72)	<0.001
Residual	-	-	-	2.36	(0.71–7.86)	0.162	0.87	(0.54–1.39)	0.561
Patient Characteristics									
Sex (ref: female)	-	-	-	1.04	(0.88–1.23)	0.622	1.14	(1.09–1.19)	<0.001
Age, y (ref: 18–49)									
50–64	-	-	-	1.04	(0.75–1.43)	0.814	0.90	(0.84–0.97)	0.004
65–79	-	-	-	1.58	(1.14–2.19)	0.006	0.77	(0.71–0.82)	<0.001
80+	-	-	-	2.72	(1.84–4.02)	<0.001	0.61	(0.55–0.68)	<0.001
Low income (ref: no)	-	-	-	0.74	(0.45–1.23)	0.251	0.93	(0.83–1.05)	0.266
In-hospital treatment									
IMA (ref: no)	-	-	-	0.31	(0.24–0.38)	<0.001	1.27	(1.16–1.38)	<0.001
Cardiopulmonary bypass (ref: no)	-	-	-	0.52	(0.41–0.66)	<0.001	0.96	(0.86–1.06)	0.389
Surgical history									
PCI (ref: no)	-	-	-	1.04	(0.85–1.26)	0.711	1.23	(1.16–1.30)	<0.001
Charlson score	-	-	-	0.96	(0.91–1.01)	0.094	0.96	(0.95–0.97)	<0.001
Medical history									
Acute myocardial infarction (ref: no)	-	-	-	1.62	(1.39–1.90)	<0.001	0.90	(0.86–0.94)	<0.001
Congestive heart failure (ref: no)	-	-	-	1.36	(1.14–1.62)	<0.001	0.85	(0.82–0.89)	<0.001
Peripheral vascular disease (ref: no)	-	-	-	1.52	(1.19–1.94)	<0.001	0.84	(0.79–0.90)	<0.001
Hypertension (ref: no)	-	-	-	2.12	(1.79–2.53)	<0.001	0.74	(0.71–0.78)	<0.001
Diabetes (ref: no)	-	-	-	0.82	(0.68–1.00)	0.046	0.93	(0.89–0.98)	0.003
Renal dysfunction (ref: no)	-	-	-	2.88	(2.36–3.51)	<0.001	0.69	(0.65–0.72)	<0.001
COPD (ref: no)	-	-	-	1.01	(0.84–1.22)	0.934	0.99	(0.94–1.04)	0.603
Traveling distance, km	-	-	-	1.00	(1.00–1.00)	0.564	1.00	(1.00–1.00)	0.552
Surgeon Characteristics									
Age, y (ref: ≦40)									
41–50	-	-	-	0.59	(0.45–0.77)	<0.001	1.16	(1.02–1.33)	0.023
51+	-	-	-	0.49	(0.31–0.77)	0.002	1.06	(0.88–1.28)	0.544
Hospital Characteristics									
Location (ref: Taipei)									
Northern	-	-	-	1.03	(0.57–1.86)	0.930	0.96	(0.83–1.11)	0.594
Central	-	-	-	0.60	(0.39–0.92)	0.019	1.96	(1.43–2.68)	<0.001
Southern	-	-	-	0.74	(0.48–1.15)	0.178	1.69	(1.37–2.07)	<0.001
Kao-Ping	-	-	-	0.77	(0.50–1.19)	0.244	1.03	(0.88–1.20)	0.732
Easten	-	-	-	1.54	(0.48–4.90)	0.469	1.05	(0.88–1.26)	0.593
Size (ref: non-large)	-	-	-	1.89	(1.11–3.22)	0.020	0.75	(0.63–0.90)	0.002
Urban (ref: rural)	-	-	-	1.79	(0.94–3.43)	0.078	0.94	(0.68–1.30)	0.719

* Regression models are adjusted for sex, age, low income, in-hospital treatment, PCI history, comorbid conditions, medical history, traveling distance, surgeon age, hospital location, size, and urban.

CI indicates confidence interval; COPD, chronic obstructive pulmonary disease; HR, hazard ratio; IMA, internal mammary artery; IV, instrumental variable; OR, odds ratio; PCI, percutaneous coronary intervention; ref, reference group.

## Discussion

This study used nationwide population-based data and applied restricted cubic spline regression and ROC curve analysis to identify optimal hospital and surgeon volume thresholds for reducing operative mortality for CABG. We also used the IV approach to explore the impact of hospital and surgeon volume thresholds on operative mortality and LOS. We found that the optimal hospital and surgeon annual volume thresholds were 55 cases and 5 cases, respectively. After adjusting for observed and unobserved covariates, we found that patients who received CABG from hospitals with previous annual volumes of < 55 cases and surgeons with previous annual volumes of < 5 cases had higher operative mortality. Patients who received CABG from low-volume surgeons had lower probability of discharge, which meant that patients receiving CABG from low-volume surgeons had longer LOS.

The rate of IMA use in Taiwan was low (70.1% between 2011 and 2015); one might expect use to be higher given IMA use is usually in the 90% range [41]. High-volume surgeons prefer to perform CABG using the greater saphenous vein; however, the rate of IMA use has increased recently. Lin et al. using Taiwan’s national health insurance research database between 1997 and 2004 have found that 20% of patients underwent CABG using the IMA [42]. In addition, the average LOS for CABG was 21.6 ± 19.9 days. Osnabrugge et al. using a multi-institutional statewide database have found that the average LOS for CABG was 6.9 ± 7.3 days in the United States [43]. The difference in LOS between Taiwan and Western countries was shown for total hip replacement [23]. The variation in the availability of resources and the organizational differences at the national and hospital level may account for the difference in LOS between Taiwan and the United States [44]. There are more hospital beds per capital with lower intensity of nurse staffing in Taiwan than in the United States. Besides, financial incentives and payment systems for hospitals and physicians may also influence LOS.

Recent evidence has explored the optimal hospital or surgeon volume threshold for various surgeries. For CABG, the Leapfrog Group, and the American College of Cardiology Foundation, and the American Heart Association have recommended hospital volume thresholds for CABG. As the number of CABG decreases, the hospital volume threshold for CABG declines from 450 cases per year to 125–150 cases per year [7]. Gutacker et al, using public hospital data from five European countries, found that patients had higher 30-day mortality if they received surgeries from hospitals with an annual volume of < 415 cases. However, they did not discover a surgeon volume threshold [7]. The apparent difference in hospital volume threshold between the present research and previous evidence could be due to the number of CABG cases [7]. Gutacker et al found that the hospital volume threshold would increase from 415 cases to 512 cases if they included data only from England and Spain, which constituted the majority of observations [7]. It is important to determine an appropriate and optimal volume threshold for improving quality of care, maintaining accessibility and facilitating healthcare resource utilization [15,23].

Both hospital and surgeon volume thresholds were related to operative mortality. Our finding was similar to that of Birkmeyer et al [2]. Based on the learning curve and practice makes perfect hypothesis, high-volume hospitals and surgeons may have more experience in managing CABG. High-volume hospitals may have experienced interdisciplinary teams, well-organized care processes, and hospital infrastructure, which are related to improve outcomes of CABG [12,45]. Moreover, surgeons who perform CABG with sufficient frequency may have accurate clinical judgment and better technical skill [2]. Our results highlight the importance of discovering optimal hospital and surgeon volume thresholds for CABG.

The surgeon volume threshold had an impact on LOS, but the hospital volume threshold did not. Our finding is similar to that of Chou and Tung [23] regarding total hip replacement and that of Aloia [46] regarding cancer surgery. Length of stay may be related to postoperative adverse events, such as infections [43]. Patients who received CABG from high-volume surgeons had lower odds of infection; however, hospital volume was not significantly associated with infection [47]. Surgeon experience has more influence on resource utilization efficiency [23,46]. As a result, identifying the surgeon volume threshold may support healthcare delivery system provide optimal care.

The volume-outcome relationship for CABG may imply that the implementation of regionalization for CABG can improve outcomes. Although the implementation of regionalization could bring benefits to patients, many patients may need to travel long distances to receive care from high-volume providers [18,48–52]. We found that the mean traveling distance for receiving CABG at the nearest high-volume hospitals was 8.8 km in Taiwan. If 30 km per hour is the safe and rational speed for urban areas in Taiwan [53], patients would take less than 30 minutes to travel to a hospital to receive CABG. Meanwhile, we found that traveling distance was not related to mortality and LOS for CABG. Recently, more studies have explored the impact of travel distance on outcomes. Similar to data in the current study, the previous studies also suggested that the benefits of receiving care at high-volume hospitals outweighed any possible travel burden [18,48–50].

In the current study, we found the nonlinear correlation of hospital and surgeon volume with risk-standardized operative mortality. We applied multivariate analysis with RCS to identify the hospital or surgeon volume corresponding to the inflection points relating to the greatest change in the log of the operative mortality. The advantages of the approach included the use of all data points to estimate the complex or linear association of hospital and surgeon volume with operative mortality and elimination of the need for prespecifying a possible threshold. Moreover, a linear correlation of hospital volume and surgeon volume with the operative mortality may not maintain at very low volume or very high volume of the model; as a result, the adoption of a RCS regression was rational than adoption of a linear regression model [22,54].

Moreover, we found that patients who received CABG from older surgeons had lower odds of operative mortality than those from younger surgeons. Provider age has been a surrogate for experience [55–57]. Previous studies have found that surgeon age was negatively related to operative mortality [58,59]. The possible mechanism is that the accumulation of skill and knowledge derived from experience could be associated with better outcomes [59].

Our study has limitations. First, the results are from Taiwan, where the number of CABG cases is much lower than in the United States and Europe, and there may be particular aspects of Taiwan’s healthcare system and patient population that may not be applied to other parts of the word. The number of CABG cases has decreased since PCI was introduced [60]. In Taiwan, more and more patients receive PCI instead of CABG. Second, we did not have information on patient-reported outcomes, so we could not explore the association of hospital and surgeon volume thresholds with these outcomes. However, a previous study found that the patient’s quality of life was related to surgical complications and LOS [61]. It is possible that hospitals with volumes that reached the thresholds and surgeons with volumes that reached the thresholds are associated with better patient-reported outcomes.

Our national population-based study showed that the hospital volume threshold was 55 cases per year and that the surgeon volume threshold was 5 cases per year. Both hospital and surgeon volume thresholds can reduce operative mortality, but only the surgeon volume threshold can improve LOS. Our study might suggest the regionalization for CABG, and our findings exhibited that the traveling distance may not influence outcomes. Moreover, determining how to ensure that surgeons reach the optimal volume threshold to increase their experience is important. For high-volume hospitals, redistributing patients within a hospital could enable some surgeons to achieve the volume threshold and prevent some surgeons from being overloaded. For small hospitals, centralizing patients to a small number of surgeons or designating a surgeon(s) to perform all CABG procedures could help surgeons achieve the volume threshold. Additionally, the optimal surgeon volume threshold can be applied in fellowship programs, which allow fellows to increase their experience with CABG procedures. Overall, it is vital to ascertain a more accurate definition of hospital and surgeon volume to help patients, providers, and policymakers deliver optimal CABG care.

## Supporting information

S1 Table Area under curve for various cut points of hospital volume. (DOCX)

S2 Table Area under curve for various cut points of surgeon volume. (DOCX)
