# Supplementary material for: Optimal surgeon and hospital volume thresholds to reduce mortality and length of stay for CABG
Source: PLoS One. 2021 Apr 14;16(4):e0249750. doi: 10.1371/journal.pone.0249750 (PMC8046183; doi:10.1371/journal.pone.0249750)
Supplement: S1 Table — (DOCX) [file pone.0249750.s001.docx]

Supplementary table 1. Area Under Curve for Various Cut Points of Hospital Volume

| Hospital volume | 40 | 45 | 50 | 55 | 60 | 65 | 70 | 75 | 80 | 85 | 90 | 95 |
| --- | --- | --- | --- | --- | --- | --- | --- | --- | --- | --- | --- | --- |
| Area under curve | 0.8188 | 0.8200 | 0.8212 | 0.8219 | 0.8208 | 0.8214 | 0.8206 | 0.8199 | 0.8201 | 0.8194 | 0.8191 | 0.8187 |
| Hospital volume | 100 | 105 | 110 | 115 | 120 | 125 | 130 | 135 | 140 | 145 | 150 | 155 |
| Area under curve | 0.8183 | 0.8183 | 0.8182 | 0.8182 | 0.8182 | 0.8182 | 0.8181 | 0.8181 | 0.8181 | 0.8181 | 0.8181 | 0.8181 |
