# Supplementary material for: Optimal surgeon and hospital volume thresholds to reduce mortality and length of stay for CABG
Source: PLoS One. 2021 Apr 14;16(4):e0249750. doi: 10.1371/journal.pone.0249750 (PMC8046183; doi:10.1371/journal.pone.0249750)
Supplement: S2 Table — (DOCX) [file pone.0249750.s002.docx]

Supplementary table 2. Area Under Curve for Various Cut Points of Surgeon Volume

| Surgeon volume | 5 | 10 | 15 | 20 | 25 | 30 | 35 | 40 | 45 | 50 | 55 | 60 |
| --- | --- | --- | --- | --- | --- | --- | --- | --- | --- | --- | --- | --- |
| Area under curve | 0.8206 | 0.8177 | 0.8170 | 0.8177 | 0.8165 | 0.8154 | 0.8149 | 0.8157 | 0.8158 | 0.8158 | 0.8152 | 0.8149 |
| Surgeon volume | 65 | 70 | 75 | 80 | 85 | 90 | 95 | 100 | 105 | 110 | 115 | 120 |
| Area under curve | 0.8165 | 0.8163 | 0.8164 | 0.8162 | 0.8162 | 0.8167 | 0.8155 | 0.8155 | 0.8161 | 0.8163 | 0.8150 | 0.8143 |
